# Pus Corpuscles in the Air; an Acroscopic Study

**Published:** 1861

**Authors:** Theoph. Eislet

**Affiliations:** Prag


### PUS CORPUSCLES IN THE AIR!

### AN AEROSCOPIC STUDY BY DOCENT DR. THEOPH. EISLET IN PRAG.

[Translated for the Boston Medical and Surgical Journal, from the Wochenblatt der Zeitschrift der k. k. Gesellschaft der Acrzte in Wein, March 26, 1861, by J. C. White, M. D.

During an epidemic of conjunctival blennorrhœa, which prevailed a short time ago in the Orphan Asylum at Repy, 8 miles distant from Prague, I had opportunity to learn by experience that infection may take place in other ways than by contact. Reserving for future description the particulars of this interesting epidemic, it will be sufficient for my present purpose to show its intensity by a few numerical data. Such foundlings as are given up by their foster-parents are brought to the large and newly-built institution at Repy. Among these 250 foundlings, of whom the majority are between the ages of 6 and 10, there occurred in 1860 from November to December forty-six, and in the period between the 16th and 21st of February, 1861, also forty-six cases of acute conjunctival blennorrhœa. His Excellency, the Governor of Bohemia, Count Forgach, presided personally on the 19th of February at a Council in Repy, at which Prof. Ritter von Hasner, Landesmedizinalrathsubstitut Dr. Hoser, Dr. Biermann, Director of the Hospital, and myself as house-physician, were present, and ordered the perfectly healthy children to be left at Repy, but the diseased and infected to be removed with the greatest haste from the institution. Forty-six children were found unaffected, while the newly attacked and those which exhibited merely an injection of the conjunctiva or papillary structure of the membrane without suppuration, were brought to Prague, and distributed in eight different localities. In the latter place, four-fifths, and in Repy, all of the children, were under my care.

It will readily be believed that as a physician I took the greatest precaution to protect myself against infection. I was particularly careful not to touch my own eyes. The cleansing of those of the patients was entrusted to the Sisters of Charity and most punctiliously performed. No chance of contagion from this source was possible, therefore, nor did any scattering of pus take place either by the patients sneezing or coughing during their examination. I was in the habit of going to the Asylum at Repy daily, where I first examined the healthy inmates, then touched the lighter cases of the disease with cuprum, and visited the worst last of all. Whenever I had spent a few hours in the wards, I was sure to feel a sensation of burning and pressure in the eyes, without being able to observe anything upon the conjunctivæ except streaks of injection on the edges of the lids. In the course of a few hours this unpleasant feeling disappeared of itself.—When the patients were brought to Prague and I visited them daily, this sensation of pain remained constant, the caruncles became red as well as the whole conjunctiva palpebrarum, and the semilunar fold became livid and so œdematous that the movements of the globe were impaired, accompanied by a mucous secretion, so that the lids adhered in the morning. In other words, I was infected without having become so by contact. The same happened without exception to all the nurses.

Of seven of the nuns severely affected, two had caught the disease by the spattering back of the water while cleaning the eyes, two from the dissemination of pus by the sneezing and coughing of the children during the same process, one by washing the bandages, and two in some inexplicable manner. The infection in my own case only needed more unfavorable circumstances to become converted into an acute affection;—as it was, however, application of weak solutions of nitrate of silver caused it to diminish in intensity.

Here, then, we have the fact that a person may be attacked by an acute conjunctival blennorrhœa without purulent contact in the ordinary sense; there is wanting only the explanation—how is this possible?

Pouchet, who for many years has been engaged in the microscopic analysis of the air, describes, in the *Compt. Rendus* April, 1860, an apparatus which he calls an aëroscope.—Through the kindness of our respected Prof. Purkyne a similar contrivance was prepared here. It depends upon the plan of driving a certain quantity of air across a glass plate moistened with glycerine, upon which the particles of dust and microscopic forms remain fixed, and may be thus readily examined by the microscope. The apparatus consists of a hydrostatic aspirator and two glass tubes, of which the first terminates at its upper extremity in a small funnel, the infundibuliform opening being directed upwards, while the lower is drawn out into a point of 0.50 of a millimetre in diameter. The second tube is ground into the first, and its upper opening is covered with a fine metallic sieve, upon which the glass plate is fastened. This plate is brought to within one millimetre's distance from the lower funnel-shaped opening by pushing in the tube, and the lower end of tube No. 2 is hermetically united to the aspirator, which is filled with water. The latter is merely a vessel made of zinc plate, two feet high and one foot square at its base, having at the bottom a stop-cock, and in the cover a mouthpiece for connection with the glass tube. If now the water be allowed to flow from the aspirator through the stop-cock, the same bulk of air will stream in through the funnel, and the matter suspended in it will remain sticking to the glass plate.

This aëroscope, as modified by Prof. Purkyne, was placed between the beds of two patients in a ward in which were thirty-three boys with acute conjunctival blennorrhœa accompanied by great secretions of pus and the air was drawn thro' it. It must, moreover, be stated that the eyes were washed by means of glass syringes with warm water, and that from this room alone several pails of waste water were thrown away daily, presenting a milky appearance from the pus it contained. The experiment was made at 10 A. M., after the apartment had been ventillated, and pus corpuscles were detected in the atmosphere by the very first transmission through the apparatus.

In this fact lies the explanation of the attacks above described, in which cases direct contact with the patients and the blennorhagic secretion was excluded. Infection took place by means of pus corpuscles suspended in the atmosphere.

---

In presenting this short but significant communication of our respected colleague and friend to the knowledge of our readers, we cannot forbear adding a few words, prompted by the importance of the subject, and with the more reason, that Dr. Eiselt has far too modestly disdained to surround the announcement of his discovery with that display, which, in science as well as in other fields of human knowledge, appears necessary to procure for a new fact its merited consideration and recognition.

The great significance of this discovery to pathology in general, and to the study of contagion in particular, and the immense importance of this fact, when more thoroughly studied and corroborated, in connection with the care of the sick and the erection of hospitals, need not be further impressed upon the physician. A new sphere of objective information is thus promisingly revealed, a new and hitherto all untrodden path opened, which, whether its results be negative or positive, will at all events lead to the advancement of science. * * * * * * * * *

We are able to say that in consideration of the high importance of this subject, many members of our society have united to give it a thorough investigation, and we shall not fail to keep our readers constantly acquainted with the progress of these examinations, which, from the abundance of suitable material afforded by Vienna, and from the combination and systematic employment of so many forces, promises a speedy and conclusive result.—*The Editors of Wochenblatt.*

